# Healthcare providers infection prevention practices and associated factors in community clinics in Bangladesh: A cross-sectional study

**DOI:** 10.1371/journal.pgph.0000574

**Published:** 2022-06-17

**Authors:** Kamrul Hsan, Md. Saiful Islam, Md. Zohurul Islam, Nurullah Awal, David Gozal, Md. Marjad Mir Kameli, Mohammad Azizur Rahman, Md. Mahfuz Hossain

**Affiliations:** 1 Department of Public Health and Informatics, Jahangirnagar University, Savar, Dhaka, Bangladesh; 2 Humanitarian Response Organization, Dhaka, Bangladesh; 3 Centre for Advanced Research Excellence in Public Health, Savar, Dhaka, Bangladesh; 4 WaterAid Bangladesh, Banani, Dhaka, Bangladesh; 5 Department of Child Health, and the Child Health Research Institute, University of Missouri School of Medicine, Columbia, Missouri, United States of America; 6 Evidence and Learnig Department, Save the Children, Cox’s Bazar, Bangladesh; 7 Department of Biochemistry and Molecular Biology, Jahangirnagar University, Savar, Dhaka, Bangladesh; Instituto Nacional de Geriatria, MEXICO

## Abstract

Healthcare associated infections impose serious challenges to safe and high-quality healthcare delivery, and have been closely associated with poor infection prevention practices. Infection prevention practices are poorly studied in Bangladesh, and no previous studies have examined these practices among healthcare providers of community clinics. The study aimed to assess infection prevention practices and associated factors among healthcare providers of community clinics in the rural area of Bangladesh. A cross-sectional study was conducted among 128 community healthcare providers in the Kurigram district of Bangladesh who were identified from 128 community clinics using a stratified random sampling technique. Data were collected between November and December, 2019 via face-to-face survey using a pre-tested semi-structured questionnaire. Only 37.5% community healthcare providers had adequate knowledge on infection prevention measures, and 39.1% had good infection prevention practices. Community healthcare providers with higher education were significantly more likely to have good infection prevention practices, and good infection prevention practices were associated with availability of hand washing facilities, and of soap in community clinic, and adequate knowledge of infection prevention. Implementation of an effective training program regarding infection prevention, along with adequate supply of infection prevention basic resources, and continuous monitoring and supervision are required to improve the currently faltering infection prevention knowledge and practices among community healthcare providers in Bangladesh.

## Introduction

Healthcare associated infections (HAIs) are the most frequent adverse events in healthcare facilities worldwide, and impose major detrimental effects on the quality of clinical services for hundreds of millions hospitalized patients every year affecting approximately 10% in developed countries and 25% in developing countries [1–5]. Indeed, the risk of HAIs is 2–20 fold higher in developing countries than in resource-rich countries, further aggravating the socio-economic burden in resource-constrained economies [6]. In Bangladesh, it is noteworthy that HAIs rates may exceed 30% in some healthcare facilities, and such high prevalence may be due to a multitude of factors, including limited resources, large number of patients and visitors, understaffing, insufficient compliance with IP and control measures, negligence in the management of hospital waste, lack of awareness of infection control problems, and last but not least, poor IP practices and knowledge among providers, all of which further reduce any motivation to implement IP measures [6, 7].

The absence of adequate IP and patient safety practices increases the risk of acquiring HAIs in both healthcare providers and patients [4, 8–10]. HAIs are important contributors to increased mortality, morbidity, antimicrobial resistance, healthcare costs for patients and their families, and lead to unnecessary prolonged hospital stays [4, 11, 12]. The World Health Organization (WHO) showed that effective implementation of IP practices in healthcare facilities leads to significant reduction (> 30%) in HAIs [13], and to annual medical cost savings of $25.0 - $31.5 billion [4, 14]. Therefore, prevention of HAIs is essential for the provision of safe and high-quality healthcare services.

The Centers for Disease Control and Prevention (CDC) and the Healthcare Infection Control Practices Advisory Committee (HICPAC) demonstrated a significant shift in healthcare delivery from the acute, inpatient hospital setting to a variety of outpatient and community-based settings over the past several decades [15]. Compared to inpatient acute care settings, outpatient and community-based settings have traditionally lacked infrastructure and resources to support IP activities [15–18]. In Bangladesh, community clinics (CCs) are the most basic primary health facilities, and were established by the government throughout the country including very inaccessible, remote, and isolated areas. About 65% of the population in Bangladesh consists of rural dwellers, and the majority are in the low socioeconomic status [19, 20]. Currently, CCs have become an integral part of the health system in Bangladesh, and aim to deliver primary health care, family planning services, maternal and child health care, nutrition and vaccination services to rural people at the grassroots level as well as execute referral link with other facilities [21, 22]. The services are maintained by mainly one CHCP for each CC [22]. So, it is important to investigate IP practices among community healthcare providers (CHCPs). However, we are unaware of any studies examining IP practices among CHCPs in Bangladesh. Therefore, we investigated IP knowledge and practices along with potentially associated factors among CHCPs in the poorest resource settings of Bangladesh that could guide the formulation and promulgation of national IP policies.

## Materials and methods

### Study design, setting and population

A cross-sectional study was conducted among CHCPs working at 128 community clinics within five Upazilas (Sub-districts) including Kurigram Sadar, Nageshwari, Bhurungamari, Phulbari and Ulipur in the Kurigram district of Bangladesh. According to the Household Income and Expenditure Survey, Bangladesh Bureau of Statistics (BBS), and World Food Programme (WFP), the Kurigram district is the poorest district in Bangladesh with a recorded 70.8% poverty rate [23, 24]. Data were collected between November and December, 2019.

### Sample size determination and sampling technique

In the study area, a total of 192 CCs were functionally active during the study period. The sample size was 128, which was calculated using the following sample size determination formula, $n=\frac{z^{2}.\;p(1-p).\;N}{e^{2}(N-1)+z^{2}.\;p(1-p)}$ considering the proportion of more frequent IP practices, 50% (since there was no previous study in the study areas), 95% confidence interval (CI) and 5% of marginal error. A stratified random sampling technique was used to select study participants. First, all community clinics in the study area were classified into five strata based on sub-district locations. Then, the CHCPs were selected from each strata using proportionate simple random sampling [25, 26].

### Data collection and quality control

Data were collected via face-to-face survey using a pre-tested semi-structured questionnaire. Approximately fifteen minutes spent for each survey. The study was formulated based on the previous studies [4, 6, 12, 27–31] and the questions were developed by a team of three experts who were knowledgeable in the area of IP. The questionnaire consisted of three sections. Section 1 comprised questions relating to socio-demographic variables (age, sex, marital status, level of education, religion, length of service.) and existing information of respondent’s community clinics (presence of IP guideline/evidence, and hand washing facility [faucet, tube well and/or basin], and availability of soap, gloves and mask). Section 2 comprised questions assessing knowledge of IP concerning IP principles, transmission of infection, hand hygiene, personal protective equipment (PPE), serialization techniques, post-exposure prophylaxis (PEP) and healthcare waste management. Section 3 included questions assessing self-reported IP practices concerning hand hygiene, use of PPE, exposure incident/needle stick injury, provision of health education about HAIs, covering of wounds, vaccination against common pathogens, and healthcare waste management.

To assure the data quality, data collection instruments were pre-tested on 13 CHCPs (10% of the intended sample size) who were drawn from the study area but not included in the actual study. The results and experiences from the pre-test were evaluated for clarity, reliability, accuracy and relevance and changes were made to the instrument by three experts who were knowledgeable in this field. The reliability coefficient for IP knowledge and practice items had a Cronbach’s Alpha value of 0.768 and 0.753 respectively. Data were examined by the principal investigators for completeness and consistency during data collection on a daily basis.

### Variables and measurements

The dependent variables evaluated were CHCP’s self-reported IP practices, whereas independent variables included socio-demographic characteristics (age, sex, marital status, level of education, religion, length of service, and history of IP training) and existing factors regarding respondent’s community clinics (IP guideline/evidence, hand washing facility, and availability of soap, gloves and masks), and knowledge of IP.

Respondents’ knowledge regarding IP and self-reported IP practices, findings were categorized using a scoring system in which, the respondent’s correct or incorrect responses to the questions were allocated “1” or “0” points respectively. The total score of knowledge questions was classified into two categories: adequate (> mean) and inadequate (≤ mean). Similarly, healthcare providers’ self-reported IP practices were classified into two categories: good (> mean) and poor (≤ mean) [12, 27, 31, 32].

### Statistical analysis

Statistical analysis relied on the Statistical Package for Social Sciences (SPSS) version 22.0. Descriptive statistics were used to calculate the frequencies, percentages, means and standard deviations of relevant variables. Chi-square tests and Fisher’s exact tests were applied to assess associations between the dependent and independent variables. In addition, binary logistic regressions were employed between dependent and independent variables and those variables with a *p-value* of less than 0.2 in the binary analysis were then entered into a multiple logistic regression to control for the effect of potential confounders. The statistical significance was declared as a *p*-value < 0.05 with a 95% of confidence interval (CI).

### Ethical considerations

This study was approved by the Biosafety, Biosecurity and Ethical Committee of the Jahangirnagar University, Savar, Dhaka-1342, Bangladesh [Ref. no: BBEC, JU/M 2020 (6)2] and all procedures were performed in accordance with the ethical standards of Institutional research ethics and Helsinki declaration. The objectives of the research were explained to the participants prior to participate in the study and written informed consent was obtained from all participants. Strict confidentiality of information and anonymity to the participants were ensured.

## Results

### Participants’ characteristics

In this study, a total of 128 CHCPs were surveyed. The average age of participants was 32.6 ± 3.7 years [SD] and half of the respondents were in the age group between 31 and 35 years old. The majority were male (57.8%) and most were Muslim (96.1%). Among the respondents, 43.8%, 33.6% and 22.7% had bachelors, masters, and higher secondary levels of education, respectively. The vast majority of the respondents’ community clinics had no IP guideline/recommendations/evidence. Only 85.9% reported that soap was always available in their community clinics. Availability of gloves and masks at all times was reported by only 13.3% and 7%, respectively (Table 1).

**Table 1:** General characteristics of the sample (n = 128).

Variables	n	(%)
**Age (years)**		
21–25	4	(3.1)
26–30	33	(25.8)
31–35	64	(50)
>35	27	(21.1)
**Sex**		
Male	74	(57.8)
Female	54	(42.2)
**Marital Status**		
Married	123	(96.1)
Single	5	(3.9)
**Education**		
Higher secondary	29	(22.7)
Bachelors	56	(43.8)
Masters	43	(33.5)
**Religion**		
Islam	123	(96.1)
Hindu	5	(3.9)
**Length of service** (years)		
<5	11	(8.6)
5–8	76	(59.4)
>8	41	(32)
**IP guideline/ evidence in CC**		
Yes	9	(7)
No	119	(93)
**Hand washing facility (tube well and/ or basin) with effective water supply in CC**		
Yes	113	(88.3)
No	15	(11.7)
**Availability of soap in CC**		
Always	110	(85.9)
Sometimes	18	(14.1)
**Availability of gloves in CC**		
Always	17	(13.3)
Sometimes	94	(73.4)
Never	17	(13.3)
**Availability of mask in CC**		
Always	9	(7)
Sometimes	27	(21.1)
Never	92	(71.9)

### Knowledge of IP

In this study, only 37.5% of the respondents had adequate IP knowledge. The average knowledge score was 5.17±1.38 [SD], with scores ranging from 2–9. Furthermore, only 42.2% of the respondents were knowledgeable that gloves cannot provide complete protection against transmission of infections and only 47.7% recognized that wearing of gloves does not replace the need for hand washing. Among the respondents, 55.5% believed that use of an alcohol-based antiseptic for hand hygiene is as effective as soap and water when hands are not visibly dirty. Furthermore, only 21.9% respondents knew how to prepare 0.5% chlorine solution, while 50% were knowledgeable that safety box should be closed/sealed when three quarters filled (Table 2).

**Table 2:** Community healthcare provider’s knowledge regarding infection prevention.

Knowledge items	n	(%)
Heard about infection prevention principles		
Yes	45	(35.2)
No	80	(62.5)
Don’t know	3	(2.3)
Gloves can provide complete protection against transmission of infections		
Yes	70	(54.7)
No	54	(42.2)
Don’t know	3	(2.3)
Washing hands with soap or use of an alcohol-based antiseptic decrease the risk of transmission of healthcare acquired infections		
Yes	119	(93)
No	7	(5.5)
Don’t know	2	(1.6)
Use of an alcohol-based antiseptic for hand hygiene is as effective as soap and water if hands are not visibly dirty		
Yes	71	(55.5)
No	57	(45.5)
Don’t know	0	(0)
W**earing of gloves replace the need for hand washing**		
Yes	61	(47.7)
No	67	(52.3)
Don’t know	0	(0)
C**hemical sterilization technique used for every equipment**		
Yes	27	(21.1)
No	95	(74.2)
Don’t know	6	(4.7)
P**hysical sterilization (heat/radiation) technique used for every equipment**		
Yes	24	(18.8)
No	93	(72.7)
Don’t know	11	(8.6)
P**ost exposure prophylaxis (PEP) for HIV after exposure**		
Yes	32	(25)
No	91	(71.1)
Don’t know	5	(3.9)
Know how to prepare 0.5% chlorine solution		
Yes	28	(21.9)
No	100	(78.1)
Should safety box be closed/sealed when three quarters filled?		
Yes	64	(50)
No	63	(49.2)
Don’t know	1	(0.8)

### Self-reported IP practices

Of those interviewed, 39.1% reported good IP practices. Moreover, only 57.9% CHCPs wash hands with soap/antiseptic before each patient care, and 86.7% wash hands with soap after patient care or contact with body fluids. The frequency of respondents who always used aprons, gloves and masks when splashes and spills of any body fluids were likely was 57.8%, 25% and 7%, respectively. In addition, 7.8% of CHCPs used IP guidelines/ evidence and 55.5% recapped needles before disposing them or preferably placing then in a safety box.

Further, 28.9% of the respondents had a preceding history of contact with blood, body fluids or needle stick injury, and among them only 24.3% underwent post-exposure prophylaxis (PEP). The majority of the CHCPs (86.7%) provided health education to healthcare recipients concerning HAIs, but only 25.8% were vaccinated against common viral pathogens. Furthermore, the majority of the CHCPs (96.1%) placed needles or sharps in safety/sharp boxes, and 55.5% disposed of the safety/sharp boxes when they were three-quarters full (Table 3).

**Table 3:** Infection prevention practice of community healthcare providers.

Practice items		n	(%)
**Wash hands with soap/antiseptic hand rub before patient care**			
Yes		74	(57.9)
No		54	(42.1)
**Wash hands with soap after patient care/contact with fluid**			
Yes		111	(86.7)
No		17	(13.3)
Always used PPE if splashes and spills of any body fluids were likely			
Apron	Yes	74	(57.8)
	No	54	(42.2)
Gloves	Yes	32	(25)
	No	96	(75)
Mask	Yes	9	(7)
	No	119	(93)
**Used infection prevention guideline/evidence**			
Yes		10	(7.8)
No		118	(92.2)
**Recap needle before disposing/placing it in safety box**			
Yes		71	(55.5)
No		57	(44.5)
**History of contact for blood, fluid or stick injury**			
Yes		37	(28.9)
No		91	(71.1)
**Measures were used after exposed for blood, fluid or stick injury (n = 37)**			
**Taking PEP**	Yes	9	(24.32)
	No	28	(75.68)
**Clean by alcohol**	Yes	10	(27.02)
	No	27	(72.98)
**Washing with water**	Yes	31	(83.79)
	No	6	(16.21)
**Provided health education to patients about HAIs**			
Yes		111	(86.7)
No		17	(13.3)
**Covered wounds on the skin before starting work**			
Yes		109	(85.2)
No		19	(14.8)
**Vaccinated against common pathogens**			
Yes		33	(25.8)
No		95	(74.2)
**Used needles or sharps put on safety/sharp boxes**			
Yes		123	(96.1)
No		5	(3.9)
**Safety/ sharp boxes disposed of when they were three-quarters full**			
Yes		71	(55.5)
No		57	(44.5)

### Factors associated with CHCPs’ self-reported IP practices

The IP practices were significantly associated with respondents’ education (χ^2^ = 8.541, df = 2, *p* = 0.014), presence of a hand washing facility (χ^2^ = 4.725, df = 1, *p* = 0.030), availability of soap in clinic setting (χ^2^ = 4.413, df = 1, *p* = 0.036) and IP knowledge (χ^2^ = 9.531, df = 1, *p* = 0.002) (Table 4).

**Table 4:** Factors associated with CHCPs infection prevention practice.

Variables	Good IP practice n (%)	Poor IP practice n (%)	χ^2^ (df)	*p*-value
**Age (years)**				
21–25	1 (0.8)	3 (2.3)	1.924 (3)	0.620
26–30	15 (11.7)	18 (14.1)		
31–35	26 (20.3)	38 (29.7)		
>35	8 (6.3)	19 (14.8)		
**Sex**				
Male	25 (19.5)	49 (38.3)	2.053 (1)	0.152
Female	25 (19.5)	29 (22.7)		
**Marital status**				
Married	48 (37.5)	75 (58.6)	0.002 (1)	1.000
Single	2 (1.6)	3 (2.3)		
**Education**				
Masters	22 (17.2)	21 (16.4)	8.541 (2)	0.014*
Bachelor	23 (18.0)	33 (25.8)		
Higher Secondary	5 (3.9)	24 (18.8)		
**Religion**				
Islam	49 (38.3)	74 (57.8)	0.794 (1)	0.648
Hindu	1 (0.8)	4 (3.1)		
**Length of service**				
<5	3 (2.3)	8 (6.3)	0.738 (2)	0.692
5–8	31 (24.2)	45 (35.2)		
>8	16 (12.5)	25 (19.5)		
**IP guideline/evidence**				
Yes	4 (3.1)	5 (3.9)	0.118 (1)	0.736
No	46 (35.9)	73 (57.0)		
**Hand washing facility (tube well and/or basin)**				
Yes	48 (37.5)	65 (50.8)	4.725 (1)	0.030*
No	2 (1.6)	13 (10.2)		
**Availability soap**				
Always	47 (36.7)	63 (49.2)	4.413 (1)	0.036*
Sometimes	3 (2.3)	15 (11.7)		
**Availability of gloves**				
Always	9 (7.0)	8 (6.3)	3.102 (2)	0.212
Sometimes	37 (28.9)	57 (44.5)		
Never	4 (3.1)	13 (10.2)		
**Availability of mask**				
Always	5 (3.9)	4 (3.1)	1.115 (2)	0.573
Sometimes	10 (7.8)	17 (13.3)		
Never	35 (27.3)	57 (44.5)		
**IP Knowledge**				
Adequate	27 (21.1)	21 (16.4)	9.531 (1)	0.002*
Inadequate	23 (18.0)	57 (44.5)		

Note

*Significant *p*-value less than 0.05.

In the unadjusted model, CHCPs who had bachelors and masters level education were three times and five times more likely to have good IP practices (COR = 3.35, 95% CI = 1.11–10.06, *p* = 0.032 and COR = 5.03, 95% CI 1.62–15.63, *p* = 0.005, respectively) (Table 5). The unadjusted model also revealed that CHCPs who had one/more hand washing facilities in CCs were 4.8 times more likely to have more frequent practices towards prevention of HAIs (COR = 4.80, 95% CI = 1.03–22.27, *p* = 0.045). Moreover, permanent availability of soap in CCs was 3.7 times more likely to result in more frequent IP practices (COR = 3.73, 95% CI = 1.02–13.63, *p* = 0.046). Furthermore, CHCPs who had more adequate knowledge about IP, were three times more likely to have more frequent IP practices (COR = 3.19, 95% CI = 1.51–6.73) (Table 5). In the adjusted model, having a master’s degree (AOR = 4.92, 95% CI = 1.41–17.23, *p* = 0.013) and adequate IP knowledge (AOR = 2.89, 95% CI = 1.26–6.63, *p* = 0.012) emerged as significant independent factors associated with more frequent IP practices (Table 5).

**Table 5:** Binary and multiple regression analysis of factors associated with infection prevention practices.

Variables	IP Practice		Unadjusted model		Adjusted model^a^	
	Good	Poor	COR (95% CI)	*p*-value	AOR (95% CI)	*p*-value
**Age**						
21–25	1 (0.8)	3 (2.3)	Reference		― ―	―
26–30	15 (11.7)	18 (14.1)	2.50 (0.24–26.60)	0.448		
31–35	26 (20.3)	38 (29.7)	2.05 (0.20–20.84)	0.543		
>35	8 (6.3)	19 (14.8)	1.26 (0.11–14.05)	0.849		
**Sex**						
Male	25 (19.5)	49 (38.3)	0.59 (0.29–1.22)	0.153	0.48 (0.20–1.14)	0.095
Female	25 (19.5)	29 (22.7)	Reference		Reference	
**Marital status**						
Married	48 (37.5)	75 (58.6)	0.96 (0.16–5.96)	0.965	― ―	―
Single	2 (1.6)	3 (2.3)	Reference			
**Education**						
Masters	22 (17.2)	21 (16.4)	5.03 (1.62–15.63)	**0.005**	4.92 (1.41–17.23)	**0.013**
Bachelor	23 (18.0)	33 (25.8)	3.35 (1.11–10.06)	**0.032**	2.66 (0.80–8.86)	0.110
Higher Secondary	5 (3.9)	24 (18.8)	Reference		Reference	
**Religion**						
Islam	49 (38.3)	74 (57.8)	2.65 (0.29–24.41)	0.390	― ―	―
Hindu	1 (0.8)	4 (3.1)	Reference			
**Length of service**						
<5	3 (2.3)	8 (6.3)	Reference		― ―	―
5–8	31 (24.2)	45 (35.2)	1.84 (0.45–7.48)	0.396		
>8	16 (12.5)	25 (19.5)	1.71 (0.39–7.41)	0.475		
**IP guideline/evidence**						
Yes	4 (3.1)	5 (3.9)	1.27 (0.32–4.97)	0.732	― ―	―
No	46 (35.9)	73 (57.0)	Reference			
**Hand washing facility (tube well and/or basin)**						
Yes	48 (37.5)	65 (50.8)	4.80 (1.03–22.27)	**0.045**	1.92 (0.37–10.06)	0.443
No	2 (1.6)	13 (10.2)	Reference		Reference	
**Availability soap**						
Always	47 (36.7)	63 (49.2)	3.73 (1.02–13.63)	**0.046**	1.93 (0.47–7.86)	0.361
Sometimes	3 (2.3)	15 (11.7)	Reference		Reference	
**Availability of gloves**						
Always	9 (7.0)	8 (6.3)	3.66 (0.84–15.93)	0.084	2.84 (0.55–14.60)	0.212
Sometimes	37 (28.9)	57 (44.5)	2.11 (0.64–6.97)	0.221	1.73 (0.45–6.59)	0.422
Never	4 (3.1)	13 (10.2)	Reference		Reference	
**Availability of mask**						
Always	5 (3.9)	4 (3.1)	2.04 (0.51–8.10)	0.313	― ―	―
Sometimes	10 (7.8)	17 (13.3)	0.96 (0.39–2.33)	0.924		
Never	35 (27.3)	57 (44.5)	Reference			
**IP knowledge**						
Adequate	27 (21.1)	21 (16.4)	3.19 (1.51–6.73)	**0.002**	2.89 (1.26–6.63)	**0.012**
Inadequate	23 (18.0)	57 (44.5)	Reference		Reference	

Notes: COR = Unadjusted/ Crude odds ratio; CI = Confidence interval; AOR = Adjusted odds ratio.

^a^Adjusted for CHCP’s sex, education, hand washing facility (tube well and/or basin), availability soap, availability of gloves, and IP knowledge.

## Discussion

This study assessed the IP knowledge and practices, and their associated factors among CHCPs in Bangladesh. The findings of the present study show that the majority of the CHCPs scored low on the IP knowledge questions (62.5%), and only 37.5% of the CHCPs had adequate knowledge about IP. The frequency of adequate knowledge is comparatively lower than several previous studies conducted among healthcare professionals in northwest Ethiopia (81.6%) [33], healthcare workers in southeast Ethiopia (53.7%) [4], and nursing staff in Kathmandu, Nepal (57.1%) [34]. There are several potential reasons behind these findings including **(i)** differences in education: most of the healthcare providers in the aforementioned studies had Diploma/Bachelor/Masters level education in Medicine or Nursing but government of Bangladesh had recruited CHCPs who had at least Higher secondary level of education in science/arts/commerce. These jobs were most suitable for individuals with Diploma of Medical Faculty (DMF), and Diploma in Nursing Sciences and Midwifery degree who studied several courss related to primary healthcare services including community medicine, public health, microbiology, etc. (all of which are essential courses to learn more about IP and infection control). However, during the recruitment of such prospective CHCPs, the government authorities were not concerned about these issue. Therefore, the lack of academic knowledge generated a significant knowledge gap on several issues of healthcare including IP; (ii) the type of healthcare staff (CHCPs *vs*. others [Doctor or Nurse]); (iii) difference in the availability and implementation of IP training; (iii) difference in the instrument to categorize IP knowledge. However, lower knowledge rates have also been reported among primary health workers in Nepal (22%) [35].

Our study found that 39.1% of CHCPs had good IP practices, a finding that is commensurate with the findings of a study conducted in Southeast Ethiopia [4]. Comparatively higher frequencies of good IP practices were however also reported by several previous studies conducted in different settings including 57.3% in Northwest Ethiopia [12], 54.2% in Bahir Dar city, Ethiopia [27], and 66.1% in Addis Ababa, Ethiopia [31]. The proportion of good IP practices among male CHCPs was higher than among female CHCPs, although there were no significant sex differences regarding overall IP practices. Notably, other studies found a higher prevalence of less frequent IP practice among male health workers [4, 12].

Not unexpectedly, IP practices were significantly associated with the level of CHCPs education, availability of hand washing facilities and soap, as well as IP knowledge. In comparison, a conducted among healthcare workers in Northwest Ethiopia found that IP practices were significantly associated with age, sex, marital status, educational status, working experience, availability of personal protective equipment and training on IP methods [12]. Similarly, another study conducted among healthcare workers in West Arsi District of Southeast Ethiopia found that IP practices were significantly associated with sex, profession, years of experience, availability of water for hand washing, the presence of an IP committee, availability of IP guidelines, and training on IP [4]. These differences may be due to differences in education status, supply of IP basic resources, sample size, socio-demographic differences, lack of in-service training and non-adherence to IP, and monitoring and evaluation system.

Although some previous studies assessed healthcare provider IP knowledge and practice, and showed associations with different factors including socio-demographic factors and IP basic resources and facilities [4, 10, 12, 18, 36], only a few studies assessed the association between healthcare provider’s IP knowledge and IP practice [37]. The present study was one of the few studies that examined the association between healthcare provider’s IP knowledge and IP practice, and found that CHCPs who had adequate IP knowledge, were three times more likely to have good IP practices than those who had no adequate IP knowledge. This finding is similar with a recent study which reported that healthcare workers who had good knowledge of infection prevention were two times more likely to have good infection prevention practices than those who had poor knowledge [37].

### Limitations

The study is not without limitations. Indeed, it is limited by the use of self-reported data, which might have influenced the findings through well-known biases (e.g., memory recall biases and social desirability biases) [38]. In addition, this study was cross-sectional in nature, and therefore cannot provide any indication of causality. Furthermore, the present study was also limited by a relatively small sample size and restricted to five sub-districts in Bangladesh, and therefore, generalizability to other sub-districts and other urban and rural regions in the country may be limited. Future studies will require longitudinal design along with expanded representative samples.

## Conclusions

We herein report important baseline information and related factors concerning several IP practices among CHCPs in Bangladesh. The majority of the respondents had limited IP knowledge and implemented IP practices less frequently. Only 39.1% CHCPs had good IP practices. Indeed, knowledge of IP, education levels, access to hand washing facilities, and availability of soap in CCs were associated with higher engagement in IP practices. Therefore, our findings suggest that healthcare authorities need to pay close attention to IP measures in CCs in order to enhance the quality of healthcare services. Implementation of an effective training program regarding IP, and fulfillment of the necessary IP resources in CCs appears to be a highly desirable, and a relatively not onerous approach that should improve IP practices among CHCPs and consequent outcomes. In addition, government and non-government stakeholders will need to ensure continuous training, monitoring and supervision to improve IP practices among CHCPs.

## Supporting information

S1 Data Data underlying the results of the study. (XLS)
